# miR‐20a/TCF4 axis‐mediated inhibition of hepatocytes proliferation impairs liver regeneration in mice PHx model by regulating CDC2 and CDC6

**DOI:** 10.1111/jcmm.16530

**Published:** 2021-05-05

**Authors:** Wei Tu, Jin Gong, Jun Song, Dean Tian, Zhijun Wang

**Affiliations:** ^1^ Division of Gastroenterology Tongji Hospital Tongji Medical College Huazhong University of Science and Technology Wuhan China; ^2^ Division of Gastroenterology Union Hospital Tongji Medical College Huazhong University of Science and Technology Wuhan China

**Keywords:** cell division cycle protein 2 (CDC2), cell division cycle protein 6(CDC6), liver regeneration, miR‐20a, transcription factor 4(TCF4)

## Abstract

MicroRNAs have emerged as essential regulators in the biological process of liver regeneration by modulating the post‐transcriptional expression of the target genes. In the present study, we found miR‐20a expression is decreased remarkably in three rodent liver regeneration models using miRNA PCR array and Venn diagram analysis. Inhibition of miR‐20a expression enhanced hepatocytes proliferation in vivo and in vitro. In contrast, overexpression of miR‐20a reduces hepatocytes proliferation and subsequently impaired liver regeneration in the mouse PHx model. Moreover, we have identified TCF4 as a target gene of miR‐20a using the PCR Array and luciferase assay. Next, mice with TCF4 deficiency were used to establish the PHx model and subjected to the examination of liver regeneration capacity. We found TCF4‐deficient mice exhibited impaired liver regeneration compared with control. Given that TCF4 acts as a transcription factor, we sort to elucidate the downstream genes involved in liver regeneration. Promoter analysis and Chip assay confirmed that TCF4 enhances CDC2 and CDC6 expression through binding to the promoter region and leads to the proliferation and cell cycle progression in hepatocytes. In conclusion, this study provides evidence that the miR20a‐TCF4‐CDC2/6 axis plays an essential role during liver regeneration.

## 1 INTRODUCTION

The healthy liver has a unique regenerative capacity after moderate injury and resection. Our understanding of the mechanism has rapidly increased over the past decades.^1^ However, a lack of regeneration in severe acute liver injury and chronic liver injury results in severe morbidity and mortality remains a clinical challenge.^2^, ^3^ Therefore, effectively stimulating liver regeneration could be a potential approach for liver failure therapy with various aetiology. In this regard, further study of the mechanism is still necessary.

MicroRNAs (miRNAs) are endogenous small non‐coding RNA molecule with approximately 22 nucleotides that functions in RNA silencing and post‐transcriptional regulation of gene expression. Abundant miRNAs have been identified to contribute to diverse biological processes in the liver, including proliferation, differentiation, tissue remodelling and cell cycle regulation.^4^, ^5^, ^6^, ^7^ Altered expression of miRNAs has been identified in the process of liver regeneration. For example, miR‐122, the most enriched miRNA in the liver, is essential for the proliferation and differentiation of hepatocytes and has been reported to promote liver inflammation during chronic liver diseases.^8^ Another study demonstrated that miR‐221 overexpression accelerates liver regeneration in mice after 2/3 partial hepatectomy by inhibiting genes that negatively regulate cell cycle progression.^9^ However, studies also showed that some miRNAs were down‐regulated during liver regeneration.^8^, ^10^, ^11^ In the present study, we found the expression of miR‐20a was significantly decreased in both mice and rat liver after PHx. miR‐20a is a member of the miR‐17‐92 cluster located at the chromosome 13q31, and is involved in various physiological and pathological processes. The functions of miR‐20a in cell proliferation remain controversial.^12^ On the one hand, some studies suggested that miR‐20a promotes cell proliferation, differentiation and tumorigenesis. For example, Liu et al demonstrated that miR‐20a‐mediated autophagy defect contributes to breast cancer onset and progression.^13^ Huang et al considered miR‐20a a biomarker to predict gastrointestinal cancers' prognosis through meta‐analysis.^14^ On the other hand, miR‐20a also has been shown to inhibit cell proliferation and act as a tumour suppressor. For example, Rui et al showed that miR‐20a is an essential negative regulator of the chondrogenic differentiation by targeting the autophagy‐related gene ATG7.^15^ Joana Marquez's group found that miR‐20a loaded nanoparticles significantly suppress colon cancer liver metastasis in mice models.^16^ Besides, accumulating data have correlated miR‐20a and liver diseases, like NAFLD, liver ischaemia, liver metabolism and hepatocellular carcinoma.^17^, ^18^, ^19^, ^20^ However, the role of miR‐20a in liver regeneration is still unknown.

In this study, we first found that miR‐20a is significantly down‐regulated in both rat and mice liver after 2/3partial hepatectomy (PHx). Moreover, overexpress of miR‐20a in hepatocytes intervenes liver regeneration in mice model of PHx, whereas silence of miR‐20a augments liver regeneration. Our findings suggest miR‐20a negatively regulates liver regeneration in the rodent PHx model. Furthermore, we demonstrated that miR‐20a post‐transcriptionally regulates TCF4 expression, which contributes to the proliferation of hepatocytes through.

## 2 MATERIALS AND METHODS

### 2.1 Animal models

#### 2.1.1 Rat PHx

Adult Male Sprague‐Dawley rats weighing 180‐200 grams were purchased from the Experimental Animal Center of Tongji Medical College, Huazhong University of Science and Technology. (Wuhan, China). Two‐thirds of hepatectomy (PHx) of the rats were performed by resection of the median and left lateral liver lobes, whereas the control rats only received transverse abdominal incision. The rats were anaesthetized with 2.5% Isoflurane (800 mL/min). The rats were killed at 24 hours, 48 hours and 168 hours after PHx.

#### 2.1.2 Mice PHx

8‐week‐old male mice were purchased from the Experimental Animal Center of Tongji Medical College, Huazhong University of Science and Technology. (Wuhan, China). After anaesthetized with 2.5% Isoflurane (300 mL/min), a transverse abdominal incision was made, the median and left lateral lobes of the liver were eviscerated subsequently. For the carbon‐tetrachloride‐induced liver injury model, mice were subjected to the intraperitoneal injection of CCl4 (0.5 mL CCl4/kg mouse bodyweight, 3:7 dilution with corn oil). Mice were killed at 24 hours, 48 hours and 168 hours after PHx with anaesthesia. All the animal experiments in this study were carried out according to the guidelines of the Animal Research Ethics Committee of Huazhong University of Science and Technology.

### 2.2 Primary mouse liver cells isolation

In situ perfusion with collagenase D(Cat No: 17104019, Thermo Fisher) and pronase E (Cat No: 10165921001, Sigma) was used to isolate liver cells from C57BL/6J mice. Mouse primary hepatocytes (PHC) were collected from the fully digested liver after centrifugation at 500 rpm. The supernatant was used to isolate primary Kupffer cells (KC) and hepatic stellate cells (HSC) by gradient Nycodenz (Cat No: AXS1002424, Axis‐Shield) density centrifugation. Liver sinusoidal endothelial cells (LSEC) were isolated from the above supernatant using the MACS method with CD31 immunomagnetic beads (Miltenyi Biotec, Cat No: 130‐097‐418). The isolated KC, HSC and LSEC were subjected to the RNA extraction and quantitative PCR. To induce the proliferation, PHC was seed on rat collagen I (Corning, Cat No:354236) coated plates with DMEM containing 10% FBS +1% Antibiotic‐Antimycotic (Cat No:15240062, Thermo Fisher) + 30 ng/mL EGF(Cat No: SRP3329, Sigma).

### 2.3 Real‐Time Quantitative PCR

Total RNA was extracted from cells and tissue using Trizol (Cat No: 15596026, Thermo Fisher) method. The concentration and purity of RNA were evaluated by the Nanodrop2000. The cDNA was prepared from 1 μg total RNA using iScript™ Reverse Transcription Supermix (Cat No: 1708841, Bio‐rad) and miScript II RT Kit (Cat No: 218161, Qiagen), for the detection of mRNA and miRNA expression, respectively.

Quantitative PCR was perform using iTaq Universal SYBR Green Supermix (Cat No: 1725120, Bio‐rad) on the Bio‐Rad CFX96 thermal cycler system. U6 snoRNA was used as an internal control for miRNA expression, and GAPDH was used for the normalization of mRNA expression. The primers used in the study were listed in Table 1.

**TABLE 1:** Primers

Mouse primers	Forward 3‐5’	Reverse 3‐5’
miR‐20a	TAAAGTGCTTATAGTGCAGGTAG	Qiagen University reverse primer CTCAACTGGTGTCGTGG AGTCGG
miR‐29c	TAGCACCATTTGAAATCGGTTAT	
miR‐374	ATATAATACAACCTGCTAAGTG	
miR‐451	AAACCGTTACCATTACTGAGTT	
U6	CTCGCTTCGGCAGCACA	
GAPDH	AGGTCGGTGTGAACGGATTTG	GGGGTCGTTGATGGCAACA
Ki67	GAGGAGAAACGCCAACCAAGAG	TTTGTCCTCGGTGGCGTTATCC
TCF4	CGCTGACAGTCAACGCATCTATG	GGAGGATTCCTGCTTGACTGTC
CDC2	AGGTACTTACGGTGTGGTGTAT	CTCGCTTTCAAGTCTGATCTTCT
CDC6	CGCAAAGTGTCTGCTGTTTCAGG	GGAGAGTGGTTTGAGGACTGTC
IL‐6	GGCGGATCGGATGTTGTGAT	GGACCCCAGACAATCGGTTG
IL‐8	GGTGAAGGCTACTGTTGG	CTGGAGTCCCGTAGAAAA
IL‐10	ACAGCCGGGAAGACAATAACT	GCAGCTCTAGGAGCATGTGG
TNF‐a	CTTCTCATTCCTGCTTGTG	ACTTGGTGGTTTGCTACG
MCP1	TTAAAAACCTGGATCGGAACCAA	GCATTAGCTTCAGATTTACGGGT
IFN‐γ	CGGCACAGTCATTGAAAGCCTA	GTTGCTGATGGCCTGATTGTC
Rat primers	Forward 3‐5’	Reverse 3‐5’
miR‐20a	TAAAGTGCTTATAGTGCAGGTAG	Qiagen University reverse primer CTCAACTGGTGTCGTGG AGTCGG
miR‐29c	TGACCGATTTCTCCTGGTGTTC	
miR‐374	ATATAATACAACCTGCTAAGTG	
miR‐451	ATGGTAATGGTTCTCTTGCTGCT	
U6	ACACTCCAGCTGGGCGCAAAT TCGTGAAGC	
GAPDH	GGCAAGTTCAACGGCACAG	CGCCAGTAGACTCCAC GAC
Ki67	ATTTCAGTTCCGCCAATCC	GGCTTCCGTCTTCATACCTAAA
TCF4	GCCAAGTCACAGACTGAGCA	GAGCGATGAGGAAGGGACCAT
CDC2	AACTGGCAGATTTCGGCCTT	GAAAAGCGGCTTCTTGGTCG
CDC6	TTAAGGCTTCCGCCCCAAAA	GGATTCCTTCTCTGGTGGGC

### 2.4 Western blotting analysis

Protein was extracted from AML12 cells using RIPA buffer (Cat No: 1708841, Sigma) according to the instruction. 30 μg protein mixed with loading buffer was fractionated by SDS‐PAGE gel and transfer to the nitrocellulose membrane. After blocking, the membrane was incubated with TCF4 (1:500 dilution, Cat No: sc‐134275, Santa Cruz) antibody at 4°C overnight and then subsequently incubated with HRP‐conjugated secondary antibody. The membrane was visualized by the enhanced ECL system. (Cat No: 32109, Thermo Fisher).

### 2.5 Immunofluorescence Staining

AML12 cells were seeded in the slide chamber (Cat No:C7182, Sigma) and treated with the mentioned stimuli, and fixed with 4% paraformaldehyde solution for 15 minutes. After blocking, cells were incubated with Ki67 (Cat No: ab15580, Abcam) antibody for 2 hours (RT, in the dark) and then incubated with Alexa Fluor 568 (Cat No: A11011, Sigma) for 30 minutes. The nucleus was stained with DAPI (Cat No: D 9542, Sigma). Images of the cells were analysed by confocal microscopy.

### 2.6 Immunohistochemistry

Paraffin‐embedded liver tissue was deparaffinized and blocked with donkey serum. Then, the slides were incubated with Ki67 antibody at 4°C overnight and HRP‐conjugated secondary antibody for 1 hour. The nuclear positive staining cells were calculated using Image J software (NIH).

### 2.7 Adenovirus and in vivo treatment

To overexpress miR‐20a in vivo, a pre‐miR20a sequence was constructed into the adenovirus vector containing a CMV promoter and EGFP reporter gene. The antagomir of miR‐20a was assembled into the adenovirus vector and used to inhibit miR‐20a expression in vivo (Genechem, Shanghai). The adenovirus vector carrying a TCF4‐shRNA sequence controlled by the CMV promoter was used to silencing the TCF4 expression in the mouse liver (Genechem, Shanghai). AD‐CMV‐U6‐EGFP was used as control. For the animal experiments, 1x109 adenovirus were injected into the tail vein of the mice after anaesthesia. The mice were used for the PHx model 2 days after tail vein injection.

### 2.8 PCR array

Liver tissues and hepatocytes were harvested for total RNA extraction using the RNeasy Mini Kit (Cat No: 74104, Qiagen). The quality of the RNA samples was evaluated by OD260/280 and OD 230/280 using NanoDrop 2000 (Thermo Fisher). In addition, the total RNA of each sample was diluted to the same concentration to facilitate the following steps. For detection of mRNA expression of the cells, the total RNA was reverse‐transcribed to cDNA using RT2 HT First Strand Kit(Cat No: 330411, Qiagen), and for the detection of microRNA expression in the liver tissue, the total RNA was reverse‐transcribed to cDNA using miScript II RT Kit. (Cat No: 218161, Qiagen). In each reaction tube, 2 μL RNA was added to 18 μL mixture containing 4 µL 5× HiSpec Buffer, 2 µL 10× Nucleics Mix, 2 µL RT Mix and 10 µL nuclease‐free water. The mixture was incubated at 37°C for 60 minutes, 95°C for 5 minutes and then cool down to 4°C. The cDNA samples were diluted 1:10 in nuclease‐free water before applying to the PCR Array. To identify the candidate microRNAs in regenerative liver tissue, we performed Mouse Liver miFinder microRNA PCR Array (Cat. No. MIMM‐116ZD, Qiagen). Mouse miR‐20a targets PCR Array (Cat. No. PAMM‐6003ZD, Qiagen) was used to identify the possible targets of miR20a in AML12 cells treated with mimic and inhibitor. In contrast, Mouse Cell Cycle PCR Array (Cat. No. PAMM‐020ZD, Qiagen) was used to find out the downstream genes of TCF4 that are involved in hepatocytes cell cycle regulation. To screen the candidate genes, miRNA (mRNA) PCR Array was performed using miScript SYBR Green PCR Kit (Cat No./: 218 076, Qiagen) on the CFX‐96 Real‐Time System(Bio‐Rad). The quantification cycle values (Cq) were calculated through the Bio‐Rad CFX Maestro Software (V4.1). Raw Cq values were uploaded to the Qiagen PCR Array tools (https://dataanalysis.qiagen.com/mirna/arrayanalysis.php) for the subsequent analysis. RNU‐6P was determined automatically as the most stable reference miRNA in the array by the tools.

### 2.9 microRNA Target validation assay

Wild‐type and mutant TCF‐4 3’UTR sequences were constructed to the pEZX‐MT05 plasmid containing dual‐luciferase reporter genes (Cat. No. HmiT054619‐MT05 and CmiT000001‐MT05, Genecopoeia). The HEK293T cells were seeded in 6‐well plates and transfected with the mentioned plasmids. 24 hours after transfection, the cells were treated with microRNA 20a mimic and control for 48 hours. Afterwards, the cell culture media was collected, and the relative luciferase activity (Gluc/SEAP ratio) was evaluated using Secrete‐Pair™ Dual Luminescence Assay Kit (Cat. No.LF033, Genecopoeia).

### 2.10 Promoter activity assay

To investigate whether TCF‐4 treatment enhances the promoter activity of CDC2/CDC6 genes through the indicated binding sites, the promoter sequences of CDC2/CDC6 (wild‐type and mutant TCF‐4‐binding sites) were constructed to the plasmids pEZX‐FR01, which contains luciferase reporter genes. Mouse primary hepatocytes were seeded in the 96‐well plates with a density of 1 × 104/well and then transfected with the pEZX‐FR01 plasmids. The cells were subsequently infected with adenovirus carrying TCF4 silence or overexpress sequences for 24 hours. The cell culture media was collected to detect the CDC2/CDC6 promoter activity by analysing the gaussian luciferase activity and secreted alkaline phosphatase activity using tSecrete‐Pair™ Dual Luminescence Assay Kit (Cat. No:LF031, Genecopeia).

### 2.11 Chromatin immunoprecipitation assay

Approximately 2 × 106 mouse primary hepatocytes were seeded in the 6cm culture dishes and infected by the adenovirus to overexpress TCF4 or silence TCF4 expression. AD‐U6‐shRNA and AD‐CMV were used as control. The Chip assay was performed by immune precipitating the DNA fragments with TCF4 antibody according to the manufacturer's instructions of CHIP kit (Cat. No:17‐371, Sigma). The anti‐mouse IgG protein was used as a negative control. The purified TCF4‐immunoprecipitated DNA was analysed by real‐time PCR using the primers specific for the promoter sequences of CDC2/CDC6 that contain different TCF4‐binding sites.

### 2.12 Statistical analyses

All data were presented as mean ± SE. The difference between the two groups was accessed by the unpaired two‐tailed Student's test. One‐way ANOVA and Tukey's post hoc analysis were used to compare the difference in multiple groups. The survival curves in the mice models were analysed using the Kaplan‐Meier method. The correlation between gene expressions was analysed by Pearson's correlation coefficient test. A *P*‐value of <.05 was considered statistically significant.

## 3 RESULTS

### 3.1 Comparison of the altered miRNA expression in mice and rat model of liver regeneration

**FIGURE 1:**
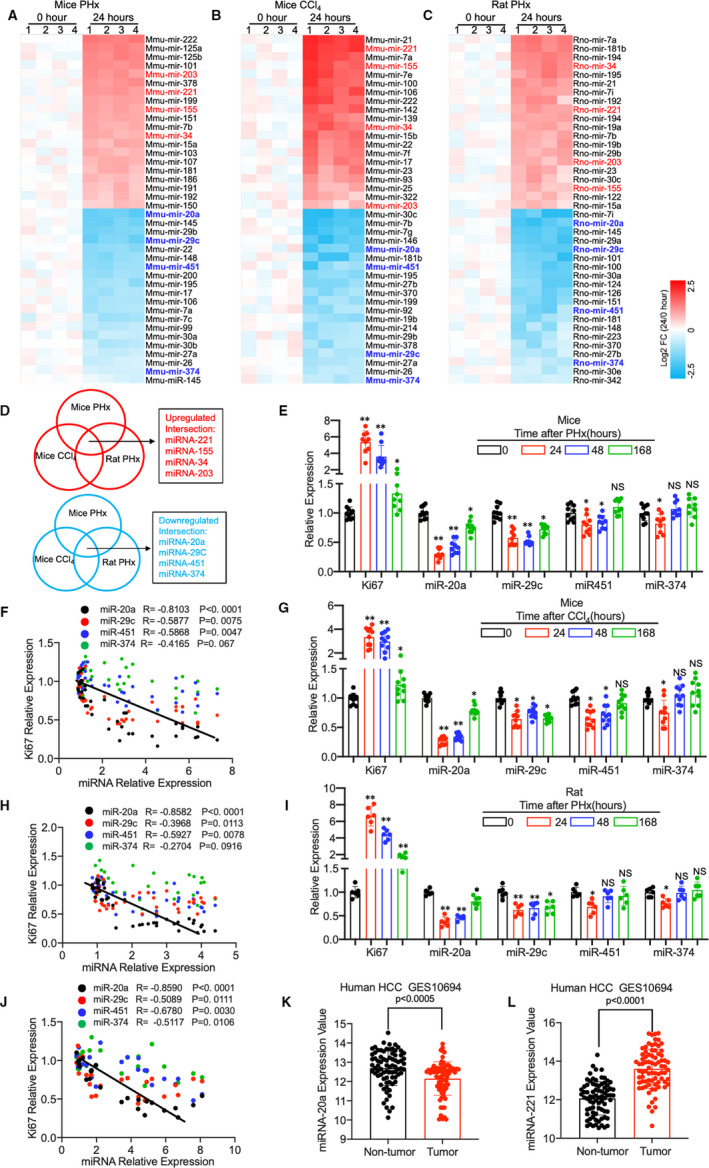
(A‐C) Heat map of the microRNA expression in mouse liver. (A) A liver disease‐related microRNA PCR Array was performed using liver tissue from mice after PHx surgery (24 h vs. 0 h, n = 3 per group). (B) MicroRNA PCR Array results of liver from mice after CCl_4_ intraperitoneal injection (Comparison between 24 h and 0 h, n = 3). (C) MicroRNA PCR Array was performed using liver from rats after PHx (24 h vs. 0 h, n = 3). (D) The Venn map shows the overlapping expression microRNAs from the three rodent liver regeneration models. (E) Relative expression of Ki67 and miR‐20a, miR‐29c, miR451 and miR374 in the liver of mice at time point 0 h, 24 h, 48 h and 168 h after PHx(0 h group n = 10, 24 h group n = 9, 48 h group n = 9 and 168 h group n = 9; GAPDH was used as a control for mRNA expression, and RNU‐6 was used to normalize microRNA expression). (F) Pearson's correlation coefficient between Ki67 mRNA expression and miR‐20a, miR‐29c, miR451 and miR374 (n = 37 per group). (G) Relative expression of Ki67 and miR‐20a, miR‐29c, miR451 and miR374 in the liver of mice at time point 0 h, 24 h, 48 h and 168 h after CCl_4_ intraperitoneal injection (n = 10 per group). (H) Pearson's correlation coefficient between Ki67 mRNA expression and miR‐20a, miR‐29c, miR451 and miR374 (n = 40 per group). (I) Relative expression of Ki67 and miR‐20a, miR‐29c, miR451 and miR374 in the liver of rat at time point 0 h, 24 h, 48 h and 168 h after PHx (n = 6 per group). (J) Pearson's correlation coefficient between Ki67 mRNA expression and miR‐20a, miR‐29c, miR451 and miR374 (n = 24 per group). (K, L) miR‐20a and miR‐221 expression in tumour tissue compared with non‐tumour tissue of GSE10694. Data are means ± SEM. *P < .05 and **P < .01

Both mice and rat PHx or CCL4‐treated model has been widely used to study the role of various molecules that modulate liver regeneration. The peak of DNA synthesis in the hepatocytes usually occurs at 24 hours in the rat after liver resection, while in mice occurs at approximately 24‐48 hours.^1^, ^2^, ^3^ Therefore, we compared the miRNA expression in mice (rat) liver 24 hours after PHx and CCl4 intraperitoneal injection to the control. Using the mouse (rat) Liver Diseases miRNA PCR Array, which included 84 miRNAs that had been confirmed to play an essential role in various liver diseases, we identified specific up‐regulated and down‐regulated miRNAs in mice and rat regenerative liver (Figure 1A,B,C). Since just one animal model cannot reflect the human disease's full features, we used the Venn map to address the overlapping miRNAs in both mice and rat liver. As shown in Figure 1D, the expression of miR‐221,^9^ miR‐155,^21^ miR‐34^22^ and miR‐203^23^ was significantly increased in both mice and rat regenerative liver, which had been reported in previous studies. For example, Yuan et al demonstrated that miR‐221 promotes liver regeneration in mice through targeting Aryl hydrocarbon nuclear translocator.^9^

Notably, we found the expression of miR‐20a, miR‐29c, miR451 and miR374 was dropped remarkably in the mice and rat liver 24 hours after administration. To our knowledge, the proliferation of hepatocytes in rodent models reaches the peak at approximately 1‐2 days and ceases within 6‐8 days after treatment.^1^, ^2^, ^3^ Therefore, we performed quantitative PCR to evaluate these four miRNAs expression at different time points, including 0 hour, 24 hours, 48 hours and 168 hours. As demonstrated in Figure 1E,G,I, miR‐20a expression showed a significant increase at 24 and 48 hours and recovered at 168 hours after PHx or CCl_4_ treatment. In contrast, the expression of miR‐29c, miR451 and miR374 was decreased at 24 and 48 hours but showed no obvious recovery at 168 hours after administration. Given that there is cell heterogeneity in the liver, we examined the expression of these miRNAs in Phx mice liver cells, including hepatocyte, Kupffer cell, hepatic stellate cell and liver sinusoidal endothelial cell, which was consistent with the results from liver tissue (Figure S1A). Next, to identify the contribution of miR‐20a, miR‐29c, miR451 and miR374 to liver regeneration, we performed Pearson's correlation analysis between the expression of miRNAs and Ki67, a common marker for hepatocyte proliferation. As shown in Figure 1E,F, we found miR‐20a is inversely correlated with the Ki67 expression remarkably compared with miR‐29c, miR451 and miR374. Consistent with the data from the mice PHx model results from the mice CCl_4_ model (Figure 1G,H) and rat PHx model (Figure 1I,J) also showed a significant correlation between the miR‐20a and Ki67 expression. Importantly, we found that compared with non‐tumour tissue, expression of miR‐20a was decreased in tumour and miR‐221 was increased by reanalysing data from human HCC GSE10694 (Figure 1K,L),^24^ which confirmed that the differential expression miRNAs screened from our PCR array are indeed essential for the hepatocyte proliferation even in human.

### 3.2 miR‐20a negatively regulates proliferation of AML12 cells

**FIGURE 2:**
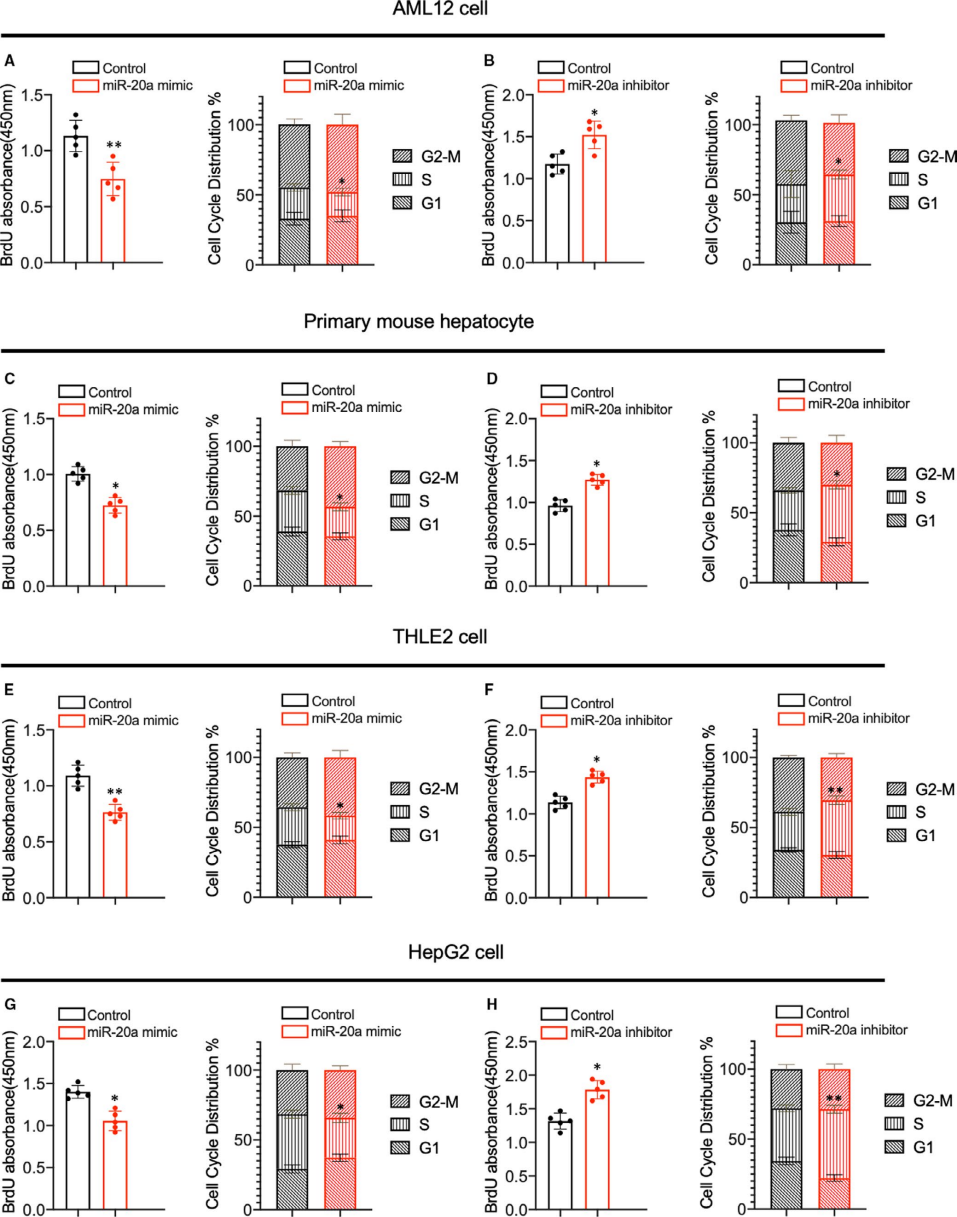
(A, B) BrdU incorporation assay and flow cytometry were used to evaluate the proliferation and cell cycle progression of AML12 cells treated with miR‐20a mimic and inhibitor (n = 5 per group). (C, D) The proliferation and cell cycle phases of primary mouse hepatocytes with miR‐20a mimic and inhibitor treatment were analysed by BrdU assay and flow cytometry (n = 5 per group). (E, F) The proliferation and cell cycle distribution of THLE2 cells treated with miR‐20a mimic and inhibitor were analysed by BrdU incorporation assay and flow cytometry (n = 5 per group). (G, H) The proliferation and cell cycle distribution of HepG2 cells treated with miR‐20a mimic and inhibitor were analysed by BrdU incorporation assay and flow cytometry (n = 5 per group). Data are means ± SEM. *P < .05 and **P < .01

The AML12 cell line was established from mouse hepatocytes, which exhibited typical hepatocyte features and was used in this study.^25^ To investigate the role of miR‐20a in hepatocyte proliferation, we transfected the AML12 cells and primary mouse hepatocyte (PHC) with miR‐20a mimic and control. After 48 hours of transfection, we found that the expression of miR‐20a was significantly increased in the mimic‐treated group compared with the control (FigureS2A, S2E). Next, we evaluated the proliferation and cell cycle phases in AML12 cells and PHC after transfection. As shown in Figure 2A,C, and Figure S2B, the proliferation, S phase and Ki67‐positive staining ratio of AML12 cells and PHC were decreased after miR‐20a mimic treatment, which indicated that miR‐20a suppresses hepatocytes proliferation in vitro. To further confirm miR‐20a negatively regulates hepatocytes proliferation, we treated AML12 cells and PHC with miR‐20a inhibitor, which down‐regulated the miR‐20a expression obviously (Figure S2C, S2F). The BrdU incorporation assay, flow cytometry analysis and Ki67 immunofluorescence staining demonstrated that proliferation, S phase (Figure 2B) and Ki67‐positive staining ratios (Figure S2D) were increased in AML12 cells with miR‐20a expression inhibition. Meanwhile, the proliferation and S phase ratio was increased in PHC after miR‐20a silencing (Figure 2D).

To investigate the role of miR‐20a on human hepatocyte proliferation, we performed overexpression or silencing of miR‐20a in the THLE2 cells and HepG2 cells. Quantitative PCR results showed that miR‐20a expression was increased after mimic treatment and decreased remarkably after inhibitor treatment in THLE2 and HepG2 cells (Figure S2G‐2J). In line with the results from mouse hepatocytes, overexpression of miR‐20a reduces proliferation and S phase in human hepatocytes (Figure 2E,H). In contrast, inhibition of miR‐20a expression augments the proliferation and cell cycle progression in THLE2 and HepG2 cells (Figure 2F,G). These findings indicated that miR‐20a inhibits the proliferation of murine and human hepatocytes.

### 3.3 Adenovirus‐mediated miR‐20a overexpression in hepatocytes delays liver regeneration in mice PHx model

**FIGURE 3:**
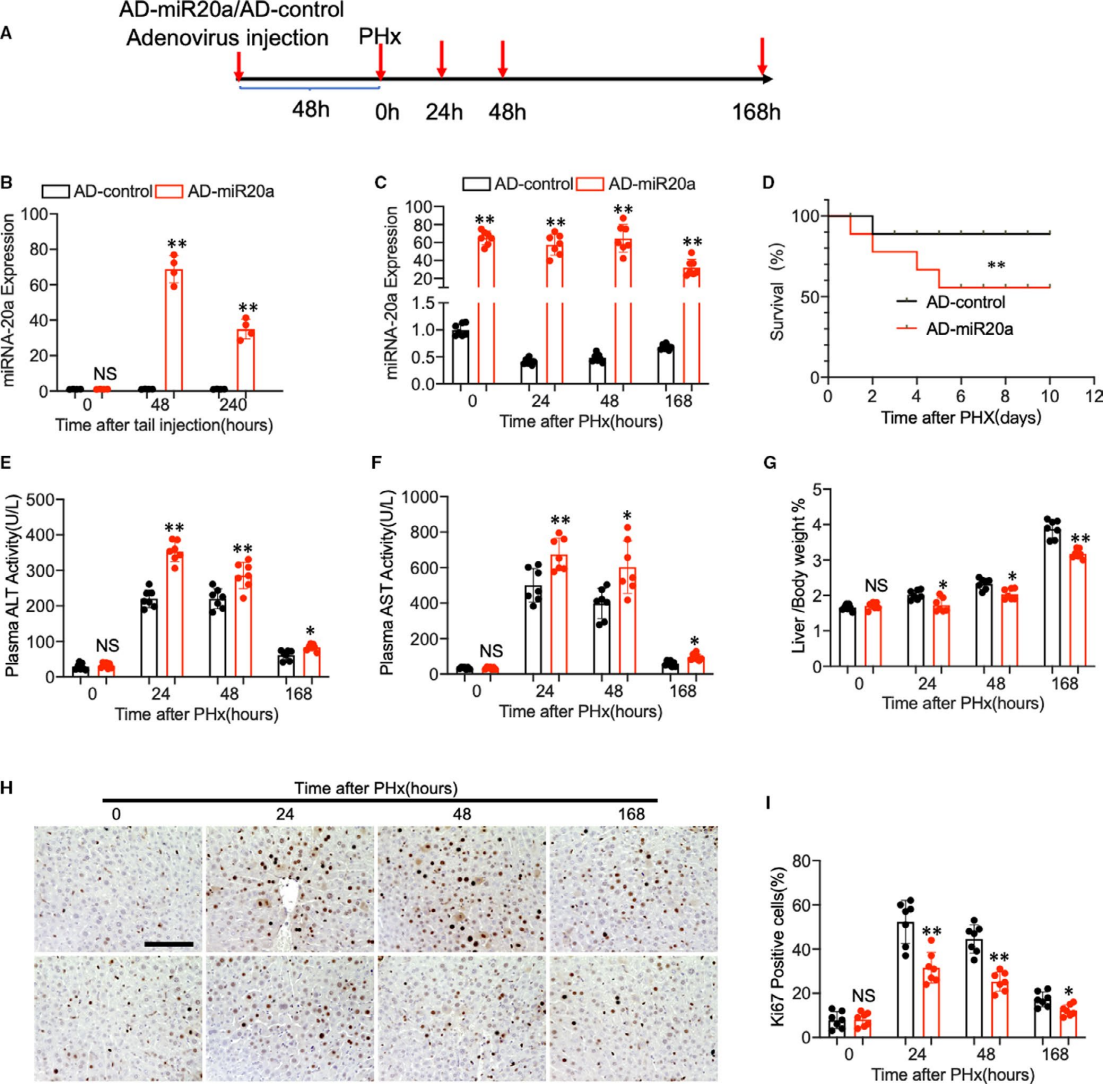
(A) Schematic diagram of mice PHx model. (B) Wild‐type C57BL/6 mice were treated with adenovirus containing miR‐20a overexpress vector through tail vein injection. miR‐20a‐relative expression in the liver was evaluated at 0 h, 48 h and 240 h after treatment (n = 4 per group). (C) Mice were subjected to the PHx surgery 48 h after adenovirus tail vein injection. Q‐PCR shows the relative expression of miR‐20a in the mouse liver at different time points (0 h, 24 h, 48 h and 168 h) after PHx (n = 7 per group). (D) Kaplan‐Meier survival curves of AD‐miR‐20a‐treated mice (n = 10) and AD‐control‐treated group. (E, F) Plasma ALT and AST levels of the mice after PHx. (G) The ratio of liver weight to bodyweight was examined. (H, I) Representative immunohistochemistry staining of Ki67 and quantitative measurement of Ki67‐positive cells ratio in mouse liver at different time points after PHx. (N = 7 per group, Scale bar, 100 μm). Data are means ± SEM. *P < .05 and **P < .01

To determine the role of miR‐20a in liver regeneration in vivo, we establish the PHx model using mice treated with an adenovirus vector carrying a pre‐miRNA‐20a expression sequence (Figure 3A). A preliminary experiment was carried out to optimize the transduction condition and efficiency of the adenovirus. As showed in Figure S3A, the GFP labelled AD‐miRNA20a vectors were successfully delivered into the hepatocytes 48 hours after tail vein injection. Besides, to further confirm that adenovirus was captured by the liver, we evaluated miR‐20a expression in PBMC and plasma. As demonstrated in Figure S3C, 3D, there was no obvious change of miR‐20a expression in PBMC and plasma 48 hours after AD‐miR20a treatment. It is well known that adenovirus treatment influences the liver immune system, which contributes to the liver regeneration process. We examined the expression of major cytokines involved in liver regeneration, including IL‐6, IL‐8, IL‐10, TNF‐a and IFN‐γ. As shown in Figure S3B, expression of IL‐6 and TNF‐a was increased in adenovirus‐treated mice liver compared with control mice. However, no significant differences were found between AD‐NC and AD‐miR20a‐treated groups.

Next, quantitative PCR indicated that miR‐20a expression was increased at the peak of 48 hours and lasted for 10 days (Figure 3B). Thus, the mice were subjected to the PHx surgery 48 hours after adenovirus tail vein injection. We found that mice injected with AD‐miRNA‐20a expressed high levels of miR‐20a at 0 hour, 24 hours, 48 hours and 168 hours after PHx compared with control (Figure 3C). Next, Kaplan‐Meier survival analysis demonstrated that miR‐20a overexpressing mice exhibited a lower survival rate than control mice (Figure 3D). In addition, miR‐20a overexpressing in the liver caused a potent increase in plasma aminotransferase (ALT) and aspartate aminotransferase (AST) level after PHx, which indicating the exacerbation of liver injury (Figure 3E,F). Moreover, miR‐20a overexpression exhibited delayed hepatocyte proliferation in vivo, evidenced by decreased liver/bodyweight ratio (Figure 3G) and Ki67 staining cells compared with control mice (Figure 3H,I). All these in vivo data indicated that miR‐20a impaired liver regeneration after PHx in the mice model.

### 3.4 Inhibition of miR‐20a expression in mice hepatocyte accelerated liver regeneration in the early stage after PHx

**FIGURE 4:**
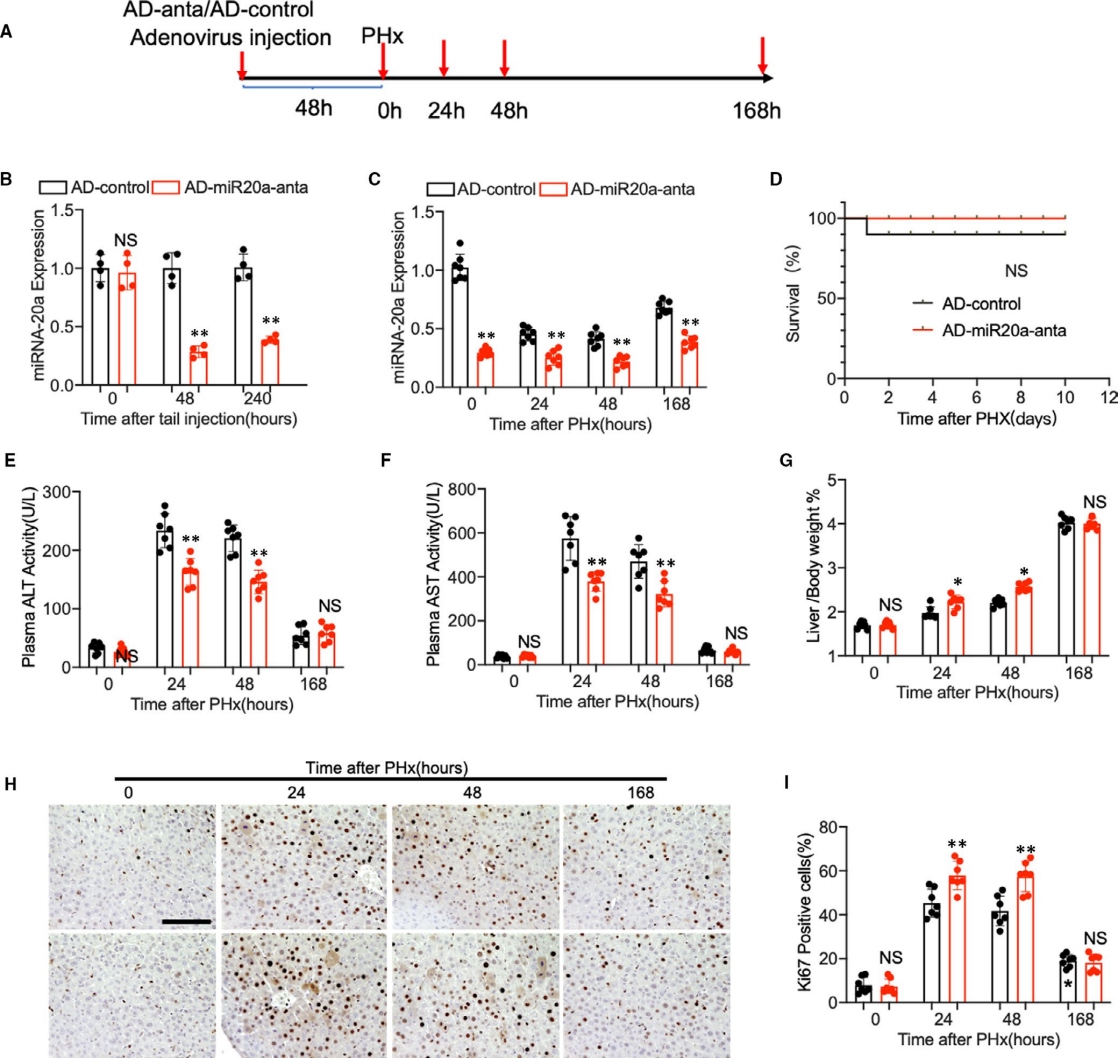
(A) Schematic diagram of PHx model using AD‐miR20a‐anta and AD‐control‐treated mice. (B) Adenovirus expressing miR‐20a antagomir and control were delivered into the mice liver through tail vein injection. miR‐20a‐relative expression in the liver was evaluated by Q‐PCR after treatment (n = 4 per group). (C) Q‐PCR shows the relative expression of miR‐20a in the AD‐miR20a‐antagomir and AD‐control‐treated mouse liver at different time points after PHx (n = 7 per group). (D) Kaplan‐Meier survival curves of AD‐miR20a‐antagomir‐treated mice (n = 10) and AD‐control‐treated group. (E, F) Plasma ALT and AST levels of the mice after PHx. (G) The ratio of liver weight to bodyweight was examined. (H, I) Representative immunohistochemistry staining of Ki67 and quantitative measurement of Ki67‐positive cells ratio in mouse liver at different time points after PHx. (N = 7 per group, Scale bar, 100 μm). Data are means ± SEM. *P < .05 and **P < .01

Given that overexpressing of miR‐20a in hepatocytes impairs liver regeneration, next, we examined the effect of miR‐20a deficiency in the regenerating liver using adenovirus expressing miR‐20a‐antagomir via tail vein injection (Figure 4A). Q‐PCR results showed that miR‐20a expression was perfectly inhibited in the liver of mice after adenovirus treatment for 48 hours (Figure 4B). When subjected to the PHx surgery, mice treated with AD‐miR‐20a‐antagomir showed a lower miR‐20a expression level than the control group during liver regeneration (Figure 4C). Nevertheless, Kaplan‐Meier survival analysis demonstrated no significant difference between the two groups (Figure 4D). Plasma ALT and AST examination (Figure 4E,F) showed that the liver injury was improved at the early stage after PHx (24 hours and 48 hours). Intriguingly, the liver/bodyweight ratio (Figure 4G) and Ki67‐positive cell ratio were higher in miR‐20a‐deficient mice at time point 24 hours and 48 hours after PHx. Still, no difference was found at 168 hours after PHx between the two groups (Figure 4H,I), which indicated that the deficiency of miR‐20a does not affect liver regeneration at the late stage.

### 3.5 TCF4 is a target of miR‐20a in the process of liver regeneration

**FIGURE 5:**
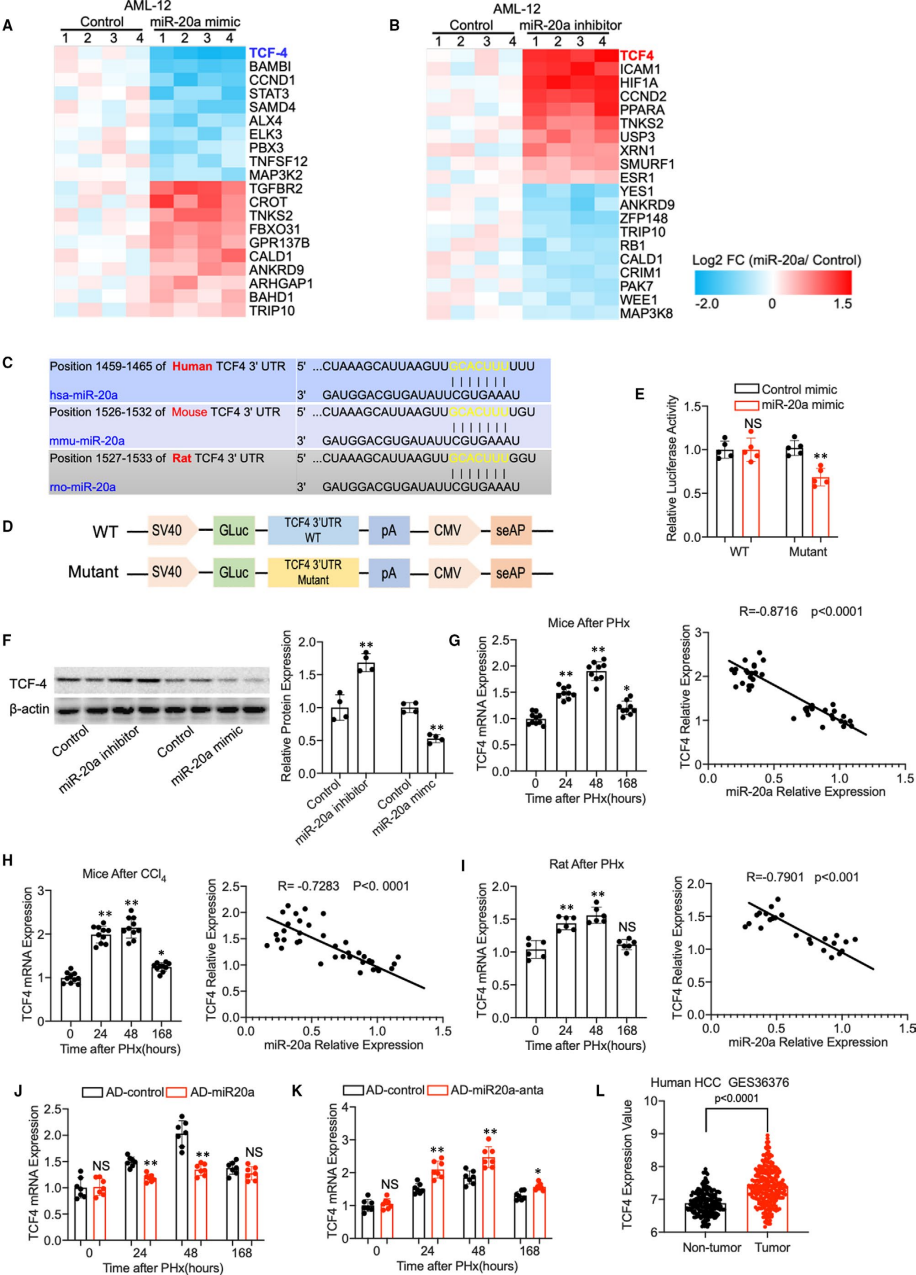
(A) A heat map of differentially expressed genes between miR‐20a mimic and control‐treated AML12 cells using miR‐20a targets PCR array (n = 4). (B) MicroRNA‐20a targets PCR array result shows the differentially expressed genes between miR‐20a inhibitor and control‐treated AML12 cells (n = 4). (C) MicroRNA‐20a‐binding sites on the TCF4 3’UTR region of human and mouse species. (D) Structure of vectors containing wild‐type (WT) and edited TCF4 3’UTR reporter. (E) Luciferase reporter assay confirms the binding of miR‐20a with 3’UTR of the TCF4 gene in the HEK293T cells (n = 5 experimental replicate). (F) Western blot shows the protein level of TCF4 in AML12 cells treated with miR‐20a mimic and inhibitor; β‐actin was used as a loading control. Quantitative measurement of TCF4 protein expression in AML12 cells (n = 4 experimental replicates). (G) TCF4 relative mRNA expression in mice liver after PHx at different time points and Pearson's correlation coefficient between TCF4 mRNA expression and miR‐20a (n = 37). (H) Q‐PCR results show the TCF4 relative mRNA expression in mice liver after CCl_4_ treatment at different time points. Pearson's correlation coefficient between TCF4 mRNA expression and miR‐20a (n = 40). (I) TCF4 relative mRNA expression in rat liver after PHx at different time points and Pearson's correlation coefficient between TCF4 mRNA expression and miR‐20a (n = 24). (J) Relative TCF4 mRNA expression in AD‐miR20a‐treated mice model of liver regeneration (n = 7 per group). (K) Relative TCF4 mRNA expression in mice model of liver regeneration with AD‐miR20a‐anta pre‐treatment. (n = 7 per group). (L) TCF4 expression in the human hepatocellular carcinoma GSE36376. Data are means ± SEM. *P < .05 and **P < .01

To elucidate the mechanism that miR‐20a regulates liver regeneration after 2/3 PHx, we used a PCR Array that includes the possible targets of miR‐20a according to the prediction of the miRNA targets database to identify the targets. Since miR‐20a inhibition promotes cell proliferation and mimics inhibits proliferation of AML12 significantly, we performed the miR‐20a targets PCR array using these samples. As shown in the heat map of Figure 5A,B, Transcription factor 4 (TCF‐4 or TCF7L2) expression was dropped remarkably in miR‐20a overexpressed hepatocytes and increased in miR‐20a‐deficient hepatocytes in contrast. Moreover, we found miR‐20a‐binding site on the TCF4 3’UTR using TargetScan analysis, suggesting TCF4 as a putative target of miR‐20a. Notably, the miR‐20a‐binding site was conserved in the TCF4 3’UTR region of humans and mice (Figure 5C). TCF‐4, also known as immunoglobulin transcription factor 2, belongs to the TCF family. Previous studies found TCF4 can form a bipartite transcription factor and influence several biological pathways, including the Wnt signalling pathway.^26^, ^27^ To further confirm that miR‐20a regulates TCF4 mRNA expression, AML12 cells were cotransfected with miR‐20a mimic and reporter plasmid containing TCF4 3’UTR region (wild type and mutant, Figure 5D). Double luciferase reporter assay showed that AML12 cells transfected with miR‐20a mimic exhibit lower relative luciferase activity in wild‐type reporters than the mutant reporter (Figure 5E), which suggested that miR‐20a binds to the TCF4 3’UTR region and induces degradation of mRNA. Besides, Western blot results showed that TCF4 protein expression was increased in AML12 cells with miR‐20a silencing and decreased when miR‐20a was overexpressed by mimic treatment (Figure 5F). All these in vitro data revealed that TCF4 is a direct target of miR‐20a in hepatocyte proliferation.

We therefore investigated the expression of TCF4 in the liver regeneration model and the correlation with miR‐20a expression. TCF4 relative mRNA expression was increased at 24 hours and 48 hours after PHx or CCl4 administration and began to decrease at 168 hours when the liver mass was restored to normal. Moreover, Pearson's correlation analysis showed that TCF4 expression was prominently related to the miR‐20a expression in the mice liver regeneration models (Figure 5G). Results from mice CCl_4_ model (Figure 5H) and rat PHx model (Figure 5I) confirmed that miR‐20a expression is correlated with TCF4 expression in rodent models of liver regeneration. We also found that TCF4 expression was decreased in miR‐20a overexpressed mice liver and increased in miR‐20a‐deficient liver (Figure 5J,K). To further prove whether TCF4 is essential for hepatocyte proliferation in humans, we analysed data from GES36376 contains over 400 cases of HCC and control. As shown in Figure 5L, TCF4 expression was significantly increased in tumour area compared with non‐tumour, which suggesting TCF4 promotes hepatocytes proliferation in the human liver. Taking together, these data indicated that TCF4 was a direct target of miR‐20a in the process of liver regeneration and may play a critical role in hepatocyte proliferation.

### 3.6 TCF4 promotes AML12 cells proliferation in vitro

**FIGURE 6:**
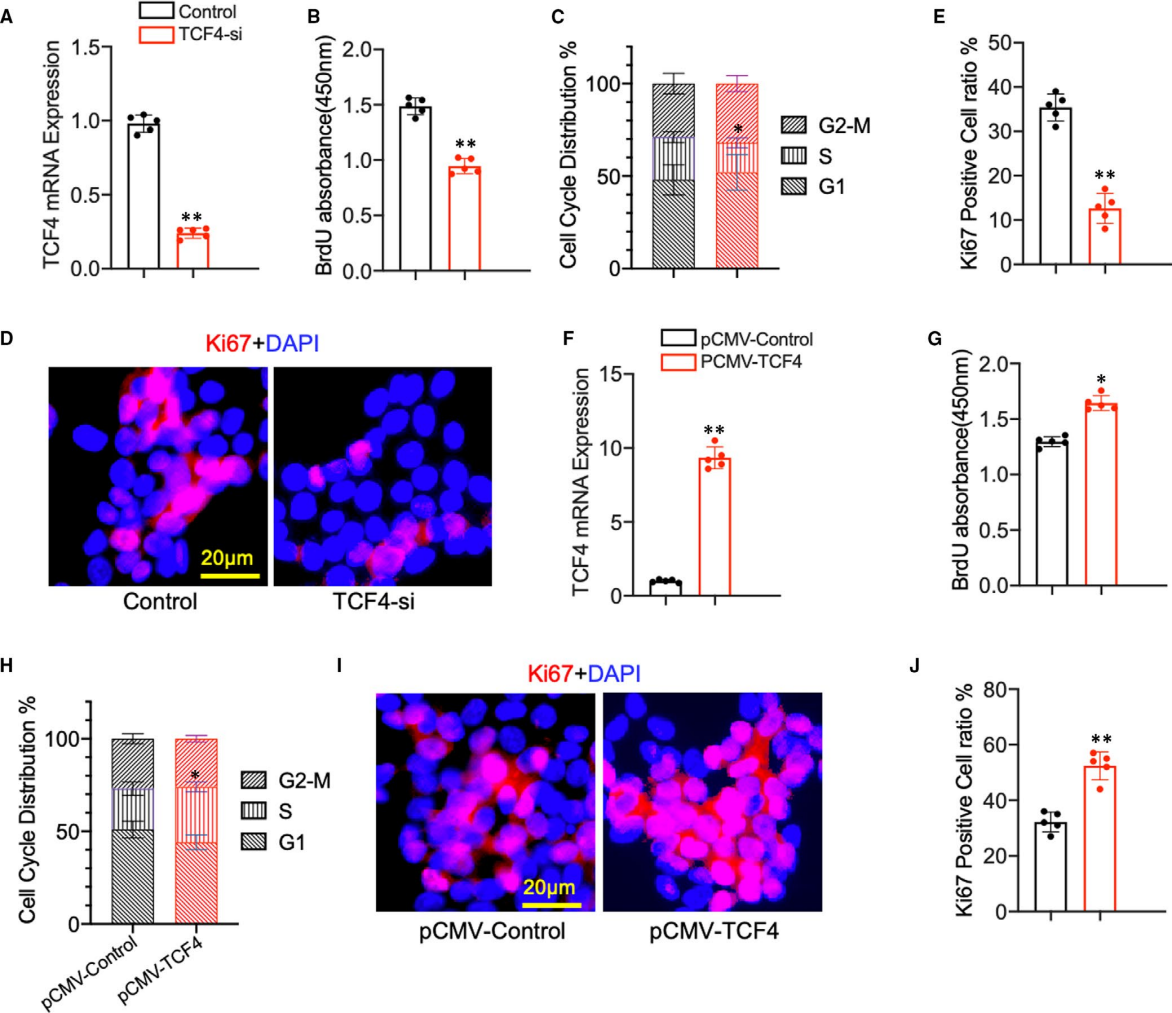
(A) Relative TCF4 mRNA expression in AML12 cells transfected with CTF4 siRNA and control (n = 5 per group ). (B) BrdU incorporation assay was used to evaluate the proliferation of AML12 cells transfected with TCF4 siRNA and control (n = 5 per group). (C) Flow cytometry analysis of the cell cycle phases in AML12 cells after TCF4 siRNA and control transfection (n = 5 per group). (D, E) Representative immunofluorescence staining images of Ki67 and quantitative measurement of Ki67‐positive cells ratio in AML12 cells. (N = 5 per group, Scale bar, 20μm). (F) Relative miR‐20a expression in AMl12 cells transfected with TCF4 overexpression and control plasmid (N = 5 per group). (G) The proliferation of AML12 cells was examined by BrdU assay (N = 5 per group). (H) Cell cycle phases were evaluated by flow cytometry (N = 5 per group). (I, J) Representative immunofluorescence staining images of Ki67 and quantitative measurement of Ki67‐positive cells ratio in TCF4 overexpressing and control AML12 cells (N = 5 per group, Scale bar, 20 μm). Data are means ± SEM. *P < .05 and **P < .01

TCF4, a member of the transcription factor family, has been reported to exert various functions within different cell types. Although previous studies showed that TCF4 promotes hepatocellular carcinoma through the Wnt pathway,^28^ the role of TCF4 in liver regeneration remains unknown. To assess the effect of TCF4 on hepatocyte proliferation, TCF4 siRNA was transfected into the AML12 cells in vitro. We found that after TCF4 expression was perfectly silenced (Figure 6A), the proliferation of AML12 was decreased remarkably using BrdU incorporation assay (Figure 6B), flow cytometry (Figure 6C) and Ki67 immunofluorescence staining (Figure 6D,E). In contrast, AML12 cells with TCF4 overexpression (Figure 6F) showed increased proliferation as confirmed by BrdU incorporation assay (Figure 6G), flow cytometry (Figure 6H) and Ki67 immunofluorescence staining (Figure 6I,J). These data suggest that TCF4 plays an essential role in normal hepatocyte proliferation.

### 3.7 Loss of TCF4 impede liver regeneration in mice after PHx

**FIGURE 7:**
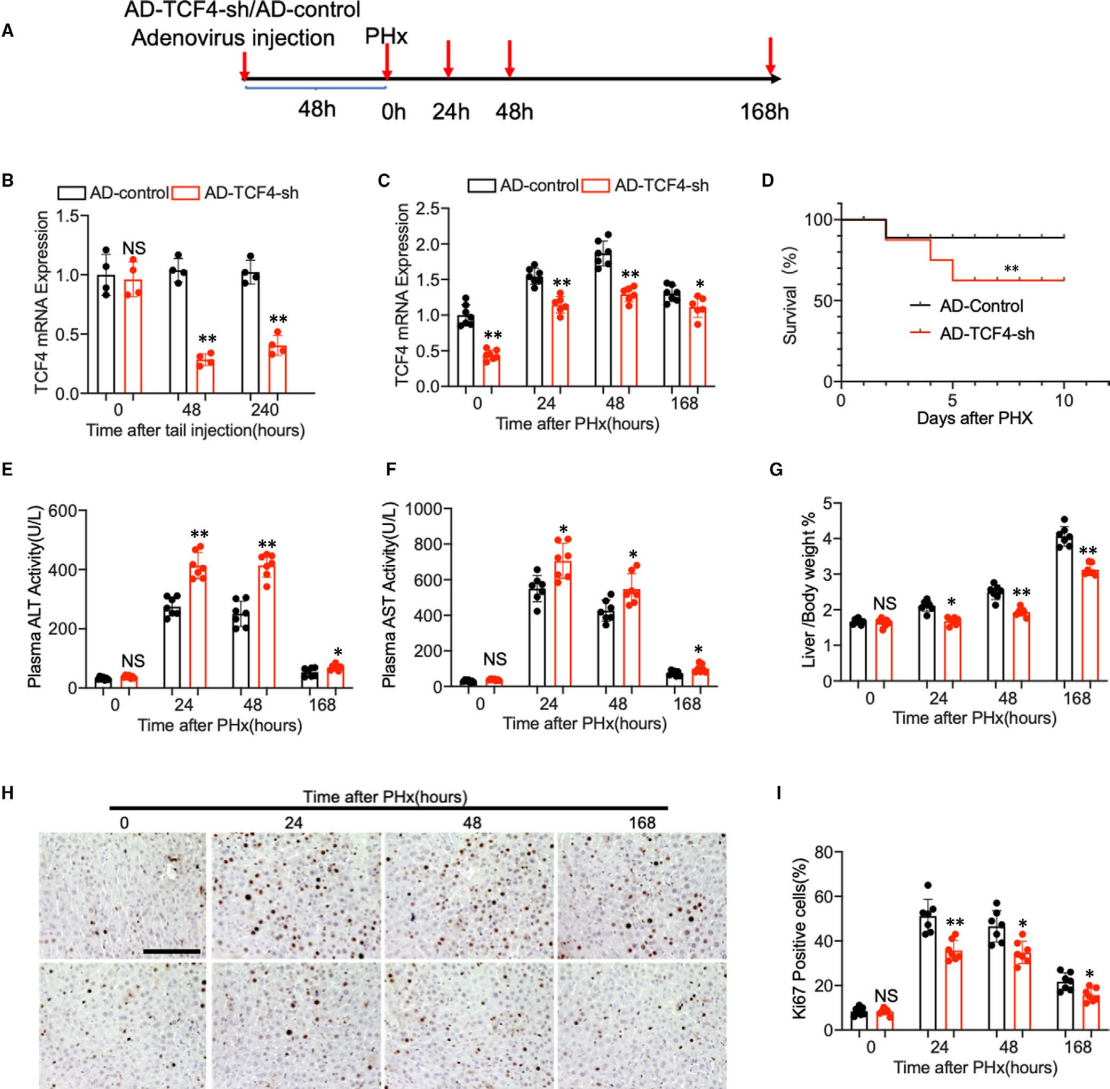
(A) Schematic diagram of mice PHx model. (B) Adenovirus carrying TCF4 shRNA and control were delivered into the mice liver through tail vein injection. TCF4 relative mRNA expression in the liver was evaluated by Q‐PCR after treatment (n = 4 per group). (C) Q‐PCR shows the relative expression of TCF4 mRNA in the adenovirus‐treated mouse liver at different time points after PHx (n = 7 per group). (D) Kaplan‐Meier survival curves of AD‐TCF4‐sh‐treated mice (n = 10) and AD‐control‐treated group. (E, F) Plasma ALT and AST levels of the mice after PHx. (G) The ratio of liver weight to bodyweight was examined. (H, I) Representative immunohistochemistry staining of Ki67 and quantitative measurement of Ki67‐positive cells ratio in mouse liver at different time points after PHx. (N = 7 per group, Scale bar, 100 μm). Data are means ± SEM. *P < .05 and **P < .01

Next, we utilized mice with TCF4 deficiency in the liver to establish the liver regeneration model (Figure 7A,B). The mice were first transduced with adenovirus carrying TCF4‐shRNA through the tail vein injection and followed by PHx treatment. As shown in Figure 7C, TCF4 expression was decreased in the AD‐TCF4‐sh‐treated group compared with the AD‐control group. Kaplan‐Meier survival curve revealed that TCF4 deficiency in the liver reduces survival rate after PHx (Figure 7D). We also found that TCF4 deficiency causes more severe liver injury in Phx mice according to the plasma ALT and AST level (Figure 7E,F). Moreover, the examination of liver/bodyweight ratio (Figure 7G) and Ki67 immunohistochemistry staining (Figure 7H,I) demonstrated that hepatocyte proliferation was reduced in TCF4‐deficient mice compared with control. Consistent with our in vitro study, these data implied that TCF4 promotes hepatocyte proliferation during liver regeneration in mice PHx model.

### 3.8 TCF4 enhances hepatocyte proliferation through the regulation of CDC2 and CDC6

**FIGURE 8:**
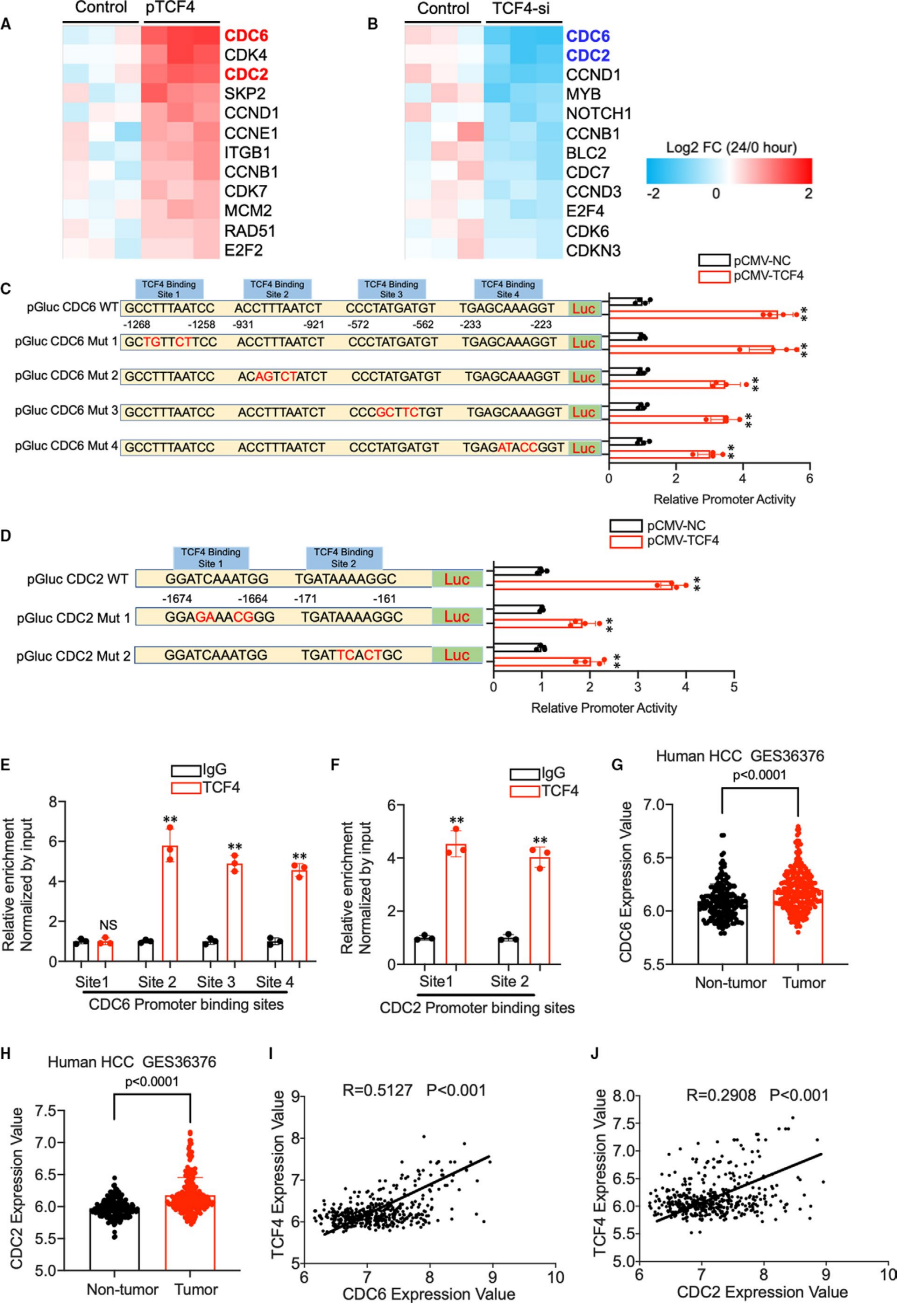
(A) A heat map of differentially expressed genes between p‐CMV‐TCF4 and p‐CMV‐Control‐treated AML12 cells using cell cycle‐related genes PCR array (n = 4). (B) PCR array result shows the differentially expressed genes between TCF4 siRNA and control‐treated AML12 cells (n = 4). (C) The promoter sequence of CDC6 contains 4 putative TCF4 transcriptional factor‐binding sites. CDC6 promoter activity was measured in HEK293T cells. (D) CDC2 promoter sequence contains 2 putative TCF4 transcriptional factor‐binding sites. CDC2 promoter activity was measured in HEK293T cells. (E, F) Quantitative analysis of ChIP experiments performed on DNA samples precipitated with antibodies against TCF4 and IgG using primers detecting CDC6 and CDC2 gene TCF4‐binding sites. (G, H) CDC2 and CDC6 expression in HCC from GSE36376. (I, J) The correlation between TCF4 expression with CDC2 and CDC6 expression in GSE36376. Data are means ± SEM. *P < .05 and **P < .01

Cell cycle‐related genes are critical regulators of hepatocyte proliferation. Although abundant pathways have been proved to be activated in liver regeneration, cell cycle genes remain the common downstream effector molecules. TCF4 is a transcription factor that affects the biological process by regulating target genes by binding to specific DNA sequences. Therefore, in this study, we screened the potential targets of TCF4 using cell cycle‐related genes array in AML12 cells with TCF4 silencing or overexpression. Intriguingly, we found CDC2 (Cell division cycle protein 2, also known as Cyclin‐dependent kinase 1, CDK1) and CDC6 (Cell division cycle protein 6) are the most significantly changed genes in AML12 cell with the intervention of TCF4 expression (Figure 8A,B). Another important finding was that the promoter region of CDC2 and CDC6 genes contains TCF4‐binding sites predicted through the PROMO analysis system. As described in the Figure, TCF4 overexpression enhanced the promoter activity level of CDC6 and CDC2 in HEK293T cells. The site‐directed mutation revealed that besides the −1268/‐1258 construct, three other TCF4‐binding sites are required for the CDC6 promoter activity; meanwhile, the two predicted TCF4‐binding sites are essential to the activity of CDC2 promoter using a luciferase reporter assay (Figure 8C,D). As expected, chromatin immunoprecipitation (ChIP) analysis demonstrated that TCF4 interacted with three of the CDC6 promoter constructs −931/‐921, −572/‐562 −233/‐223, and also interacted with both CDC2 promoter constructs −1674/‐1664, −171/‐161 (Figure 8E,F). Next, we provide evidence that CDC6 and CDC2 are critical for hepatocyte proliferation in vivo. Q‐PCR results revealed that CDC6 and CDC2 mRNA expressions increased dramatically in the liver of rodent models after PHx or CCl4 administration, as showed in Figure 8G,H. Surprisingly, we found CDC2 and CDC6 expression was significantly increased in human HCC samples compared with non‐tumour tissue (Figure 8I). Furthermore, the expression of CDC2 and CDC6 are strongly related to TCF4 expression in human HCC samples from GES36376 (Figure 8J). These results indicated that the TCF4‐CDC2/CDC6 axis enhances liver regeneration in rodents and is important in human hepatocyte proliferation.

## 4 DISCUSSION

The normal adult liver is mitotically quiescent and undergoes a rapid alteration to restore the primary mass, structure, and functions following injury and resection. Rodent models of liver regeneration after PHx or CCL4 administration have greatly increased our understanding of the mechanisms that drive the regenerative process. To date, various molecules and pathways were discovered to regulate cell proliferation during liver regeneration, including miRNAs. In this study, we compared the differential expression miRNAs among three rodent liver regeneration models and identified certain overlapping miRNAs, especially miR20a. miR‐20a belongs to the miR‐17‐92 family which including miR‐17‐5p, miR‐17‐3p, miR‐18, miR‐19a, miR‐20a, miR‐19b‐1 and miR‐92‐1. Previous studies indicated that miR‐20a was involved in multiple biological processes, such as immune response, vascular remodelling, cell differentiation and tumorigenesis.^29^, ^30^, ^31^, ^32^ The role of miR‐20a in tumorigenesis is controversial, as it was considered an oncogene in colorectal cancer but a tumour suppressor in breast cancer and hepatocellular carcinoma. Several studies showed that miR‐20a suppresses hepatocellular carcinoma onset and metastasis by down‐regulating oncogenes and related pathways.^33^, ^34^ Together with our miRNA PCR Array comparison results from three rodent models, we hypothesize that miR‐20a plays an essential role in liver regeneration.

To investigate whether miR‐20a promotes hepatocytes proliferation, we performed in vitro experiments using AML12 cells with miR‐20a inhibition or overexpression. As expected, miR‐20a overexpression reduces AML12 cell proliferation. In contrast, miR‐20a inhibition enhances AML12 cell proliferation. Consistently with the in vitro results, miR‐20a overexpression in the liver using adenovirus carrying pre‐miR‐20a impaired liver regeneration in mice after PHx. Meanwhile, mice with miR‐20a deficiency exhibit accelerated liver regeneration at an early stage after PHx. However, on day 7 after PHx, no significant difference was found between miR‐20a‐deficient and control mice. To our knowledge, the proliferation of hepatocytes ceases within 6‐8 days after resection, when the liver restores to approximately 85% of the normal mass. Multiple molecules and signallings were proven to be involved in the termination of liver regeneration.^2^, ^3^ For example, integrin‐linked kinase (ILK) secreted by hepatic stellate cells and hepatocytes suppresses hepatocytes proliferation through interacting with YAP signalling.^35^ The expression of ILK was induced at the late stage of liver regeneration. ILK‐deficient mice after PHx develop larger liver size, up to 158% of the normal liver.^36^ Prior studies revealed that GPC3 inhibits YAP and downstream genes by activating the Hippo pathway.^37^, ^38^ WNT5A has also been found to impede the WNT/β‐catenin signalling pathway and leads to the termination of liver regeneration in mice.^39^ In general, as liver mass gradually restored, multiple gene expression were changed and eventually leading to the suppression of hepatocytes proliferation. Based on these observations, we speculated that despite the miR‐20a expression was inhibited by the adenovirus at the end stage of liver regeneration, the signalling that controls normal liver size neutralizes the effect of miR‐20a deficiency on hepatocytes proliferation. These compensatory pathways, including Hippo‐Yap and WNT/β‐catenin, prevent the liver from exceeding growth.

According to these data, we confirmed that miRNA‐20a plays an important role in mouse liver regeneration in mice after PHx. It is well known that miRNAs are involved in virtually every cellular process by the silencing of target genes. Theoretically, just one miRNA can regulate hundreds of target genes through binding in the 3′ untranslated region (UTR) of mRNAs. However, the genes targeted by the miRNA can differ significantly for different cells and diseases.

In this study, we have identified several putative targets like TCF4, CCND1 and STAT3 in hepatocytes proliferation using a PCR Array, including all the predicted target genes of miR‐20a. CCND1 was a widely studied cyclin that promotes the cell cycle progression, and play an important role in liver regeneration of mouse model.^40^ The activation of STAT3 pathway by various cytokines and growth factors, including IL‐6, IL‐22 and EGF, also contributes to the hepatocyte proliferation in liver regeneration.^41^, ^42^ However, the role of TCF4 on liver regeneration remains unclear. TCF4 is an important transcription factor that exhibits diversified functions in different cell types and disease models. For instance, in the central nervous system, TCF4 controls the cholesterol biosynthesis during oligodendrocyte development^43^ and regulates the stimulatory actions of nicotine on a habenula‐pancreas axis.^44^ Besides, TCF4 is essential for glucose metabolism in many tissues such as the gut, brain, liver and skeletal muscle.^45^, ^46^ As demonstrated in some studies, TCF4 also contributed to various cancer growth and metastasis, including lymphoma,^47^ prostate cancer,^48^ ovarian cancer,^49^ colorectal cancer^50^, ^51^ and hepatocellular carcinoma. However, the function of TCF4 in liver regeneration remains unclear. To this end, we first evaluated the role of TCF4 in AML12 cells. As expected, silencing of TCF4 expression reduces AML12 cells proliferation, and overexpression of TCF4 in AML12 cells promotes proliferation in contrast. Moreover, in vivo results showed that inhibition of TCF4 expression in the liver significantly impairs the regenerative process in mice PHx model.

TCF4 is a transcription factor that regulates cellular processes through binding to the promoter region of the downstream genes. Previous studies revealed that TCF4 promotes hepatocellular carcinoma progression through various molecules and signalling.^52^, ^53^, ^54^ In accordance with zhang C’s research, TCF4 can bind to the AJUBA promoter regions and leads to the activation of the Akt/GSK‐3β/Snail pathway, which drives the EMT progression in HCC.^28^ Blockade of TCF4/Wnt pathway using a small molecule antagonist suppress the proliferation of the cancer cells in vivo and vitro through targeting c‐Myc, cyclin D1 and surviving.^55^ A study from Yoshito's group demonstrated that a novel drug, C‐122, inhibits tumour growth in hepatocellular carcinoma through down‐regulation of TCF4 and downstream genes such as SPP1, AXIN2, MMP7, ASPH, CD24, ANXA1 and CAMK2N1.^56^ Cell cycle‐related genes are the essential regulators in the process of liver regeneration. Despite the fact that various signalling will be activated in the liver after injury or resection, eventually, they will lead to the alteration of cell cycle‐related genes. Therefore, we conducted the cell‐cycle‐genes specific PCR array in the AMl12 cells with TCF4 silencing and overexpression. The results showed that when TCF4 expression is inhibited, the cell cycle proteins CDC2 and CDC6 decreased the most significantly, while they increased dramatically when TCF4 is overexpressed.

Previous studies have shown that CDC2 and CDC6 play an essential role in promoting cell cycle progression.^57^, ^58^, ^59^, ^60^ We further discovered using promoter‐binding site analysis software (JASPAR and PROMO) that the promoter region of CDC2 has two TCF4‐binding sites and that of CDC6 has four TCF4‐binding sites. This suggests that TCF4 may regulate the expressions of CDC2 and CDC6 by binding to their promoter regions and thus stimulate cell cycle progression in hepatocytes. Importantly, we found that expression of TCF4, CDC2 and CDC6 is up‐regulated in HCC tumour compared with non‐tumour tissue from GSE 37367, including over 400 human HCC samples. Moreover, Pearson's correlation analysis revealed that the TCF4 expression was strongly related to CDC2 and CDC6 expression, which indicated that the TCF4‐CDC6/CDC2 regulation axis was also pivotal for human hepatocyte proliferation.

However, there are limitations to this study. First, the animal models and Venn diagram analysis we used were not ideal since physiological processes and underlying mechanisms of three rodent liver regeneration models were not identical. Second, some of the microRNAs included in the miRNA PCR Array were not conserved across different species.

The miRNAs screened from the three rodent models may exert different functions in humans, mice and rats. Third, a long‐term stable inhibition of miR‐20a in hepatocytes may lead to the overexpression of TCF4, which, in turn, causes tumorigenesis of the liver.

Therefore, to avoid the increased risk of HCC, we need to develop appropriate approaches that provide a balance between hepatocyte proliferation and non‐tumorigenicity. In other words, we have to control the duration of target gene expression in the liver precisely. For example, adenovirus or AAV mediated transient modification of gene expression in hepatocytes. Besides, recent studies demonstrated that exosome can served as a potential transport cargo in the treatment of various disease,^61^, ^62^, ^63^ including liver disease. However, there are important distinctions between basic research and translational medicine. Thus, as the fundamental of the clinical applications, mechanistic studies in liver regeneration are still necessary.

In summary, this study provides evidence that miR‐20a reduces mice liver regeneration after PHx by down‐regulating the expression of TCF4 and consequently leads to the inhibition of cell cycle progression through CDC2 and CDC6. Therefore, based on our findings, a strategy aiming at modulating the miR20a‐TCF4‐CDC6/CDC2 regulation axis may be beneficial for liver regeneration in the clinic.

## CONFLICT OF INTEREST

The authors confirm that there are no conflicts of interest.

## AUTHOR CONTRIBUTION

**Wei Tu:** Data curation (lead); Formal analysis (lead); Investigation (lead); Methodology (lead); Project administration (lead). **Jin Gong:** Data curation (supporting); Formal analysis (supporting); Investigation (supporting); Methodology (supporting); Project administration (supporting). **Jun Song:** Conceptualization (supporting); Supervision (supporting). **De‐An Tian:** Conceptualization (supporting); Funding acquisition (supporting); Writing‐review & editing (supporting). **Zhijun Wang:** Conceptualization (lead); Funding acquisition (lead); Writing‐original draft (lead); Writing‐review & editing (lead).

## Supporting information

Fig S1

Fig S2

Fig S3
