# Supplementary figures and images for: miR‐20a/TCF4 axis‐mediated inhibition of hepatocytes proliferation impairs liver regeneration in mice PHx model by regulating CDC2 and CDC6
Source: J Cell Mol Med. 2021 May 5;25(11):5220–37. doi: 10.1111/jcmm.16530 (PMC8178283; doi:10.1111/jcmm.16530)

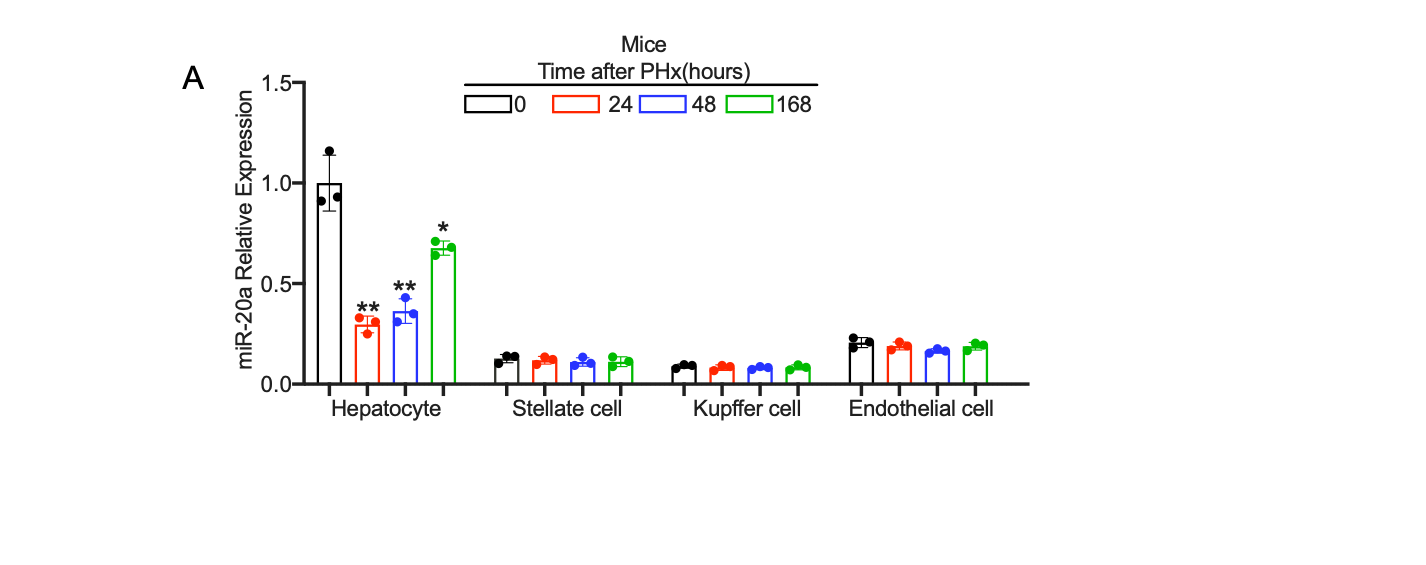

Supplement: Supplementary file 1 — Fig S1 [file JCMM-25-5220-s003.tiff]

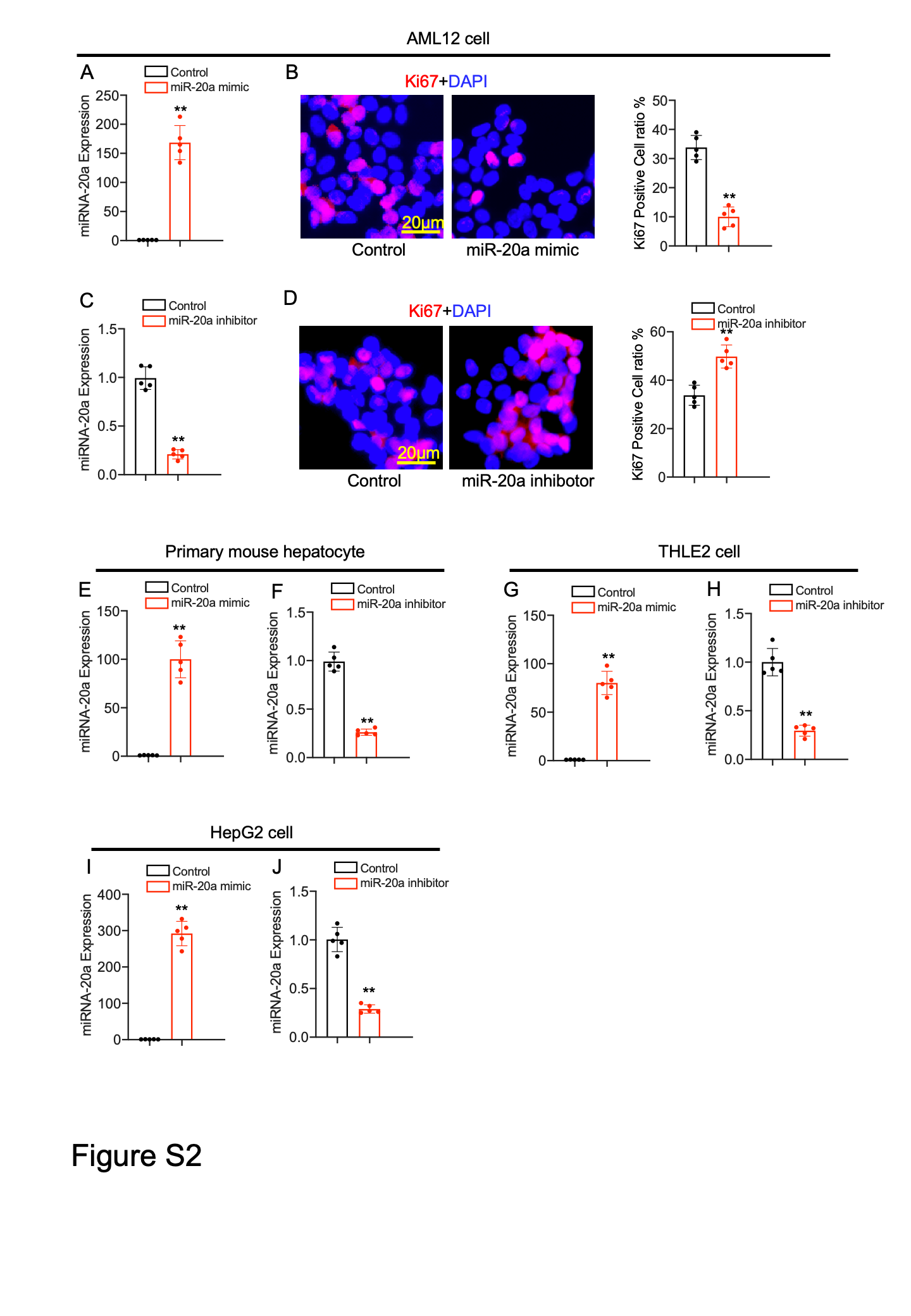

Supplement: Supplementary file 2 — Fig S2 [file JCMM-25-5220-s001.tiff]

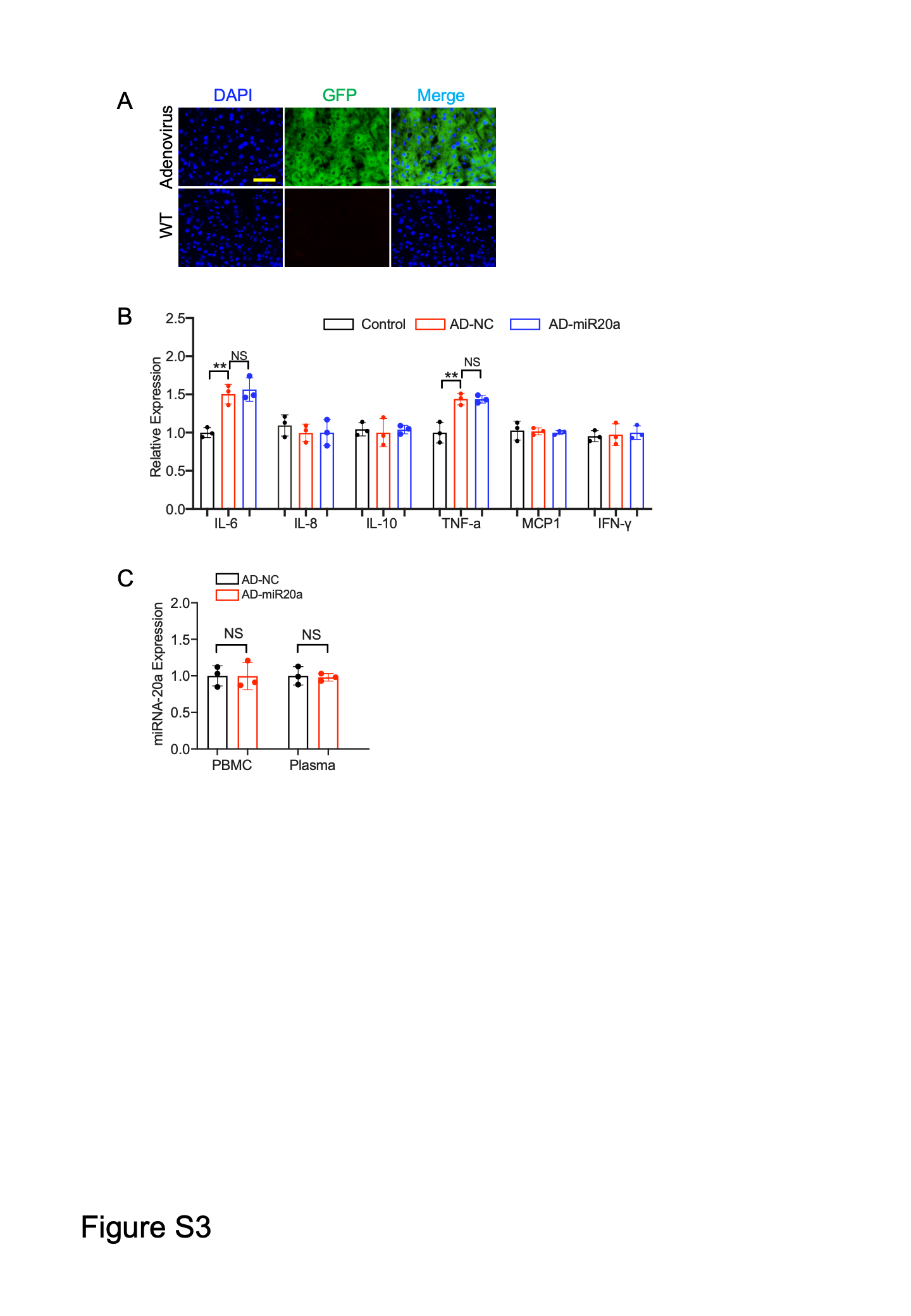

Supplement: Supplementary file 3 — Fig S3 [file JCMM-25-5220-s002.tiff]
